# Prognostic value of hematologic parameters in advanced non-small cell lung cancer patients receiving anti-PD-1 inhibitors

**DOI:** 10.3389/fimmu.2022.1003581

**Published:** 2022-10-20

**Authors:** Xinmin Zhao, Xianghua Wu, Hui Yu, Huijie Wang, Si Sun, Zhihuang Hu, Cuicui Liu, Junli Zhang, Yang Shao, Jialei Wang

**Affiliations:** ^1^ Department of Thoracic Medical Oncology, Fudan University Shanghai Cancer Center, Shanghai, China; ^2^ Department of Oncology, Shanghai Medical College, Fudan University, Shanghai, China; ^3^ Geneseeq Research Institute, Nanjing Geneseeq Technology Inc., Nanjing, Jiangsu, China; ^4^ School of Public Health, Nanjing Medical University, Nanjing, Jiangsu, China

**Keywords:** hematologic parameters, prognosis, first-line pembrolizumab, subsequent-line, nivolumab, NSCLC

## Abstract

**Background:**

The association between hematologic parameters and anti-programmed death-1 (PD-1) inhibitors was generally examined without considering therapy lines and medicine types. The study was aimed to identify potential hematologic biomarkers associated with clinical outcome in patients with non-small cell lung cancer (NSCLC) treated with first-line pembrolizumab and subsequent-line nivolumab.

**Materials and methods:**

161 NSCLC patients were categorized into first-line pembrolizumab group (pembrolizumab group) and subsequent-line nivolumab group (nivolumab group). Univariate and multivariate Cox regression analyses were used to evaluate the prognostic value of hematologic parameters for clinical outcomes.

**Results:**

The median progression-free survival (mPFS) was 9.6 months in the pembrolizumab group and 4.1 months in the nivolumab group (HR =1.61; P = 0.012); the median overall survival (mOS) was not reached in the pembrolizumab group and 17.7 months in the nivolumab group (HR =1.37; P = 0.23). Of the 79 patients in the pembrolizumab group, baseline PD-L1 tumor proportion score (TPS)≥1% was an independent factor of longer PFS and OS. Age≥60 years, absolute platelet count (APC)≥220×10^9^/L and platelet-to-lymphocyte ratio (PLR)≥120 were associated with inferior PFS. Of the 82 patients in the nivolumab group, absolute neutrophil count (ANC)≥3×10^9^/L was associated with longer PFS, while LDH (lactate dehydrogenase)≥160 U/L was associated with inferior PFS and derived neutrophil-to-lymphocyte ratio (dNLR)≥1.2 was associated with longer OS.

**Conclusion:**

Our study identified multiple clinically accessible prognostic biomarkers in the peripheral blood in both the pembrolizumab and nivolumab subgroups.

## Introduction

Lung cancer is the leading cause of cancer death in the world and non-small cell lung cancer (NSCLC) accounts for more than 80% of all lung cancer cases (1, 2). For patients who lack targetable driver alterations, immune checkpoint inhibitors targeting programmed cell death 1 (PD-1) have demonstrated better efficacy than chemotherapy (3–5). Nivolumab and pembrolizumab are the first two anti-PD-1 inhibitors that have received US Food and Drug Administration (FDA) approval (6). From 2018 to 2020, combination therapy of pembrolizumab plus chemotherapy and nivolumab monotherapy have been approved as first-line and second-line therapies, respectively, for NSCLC patients in China. PD-1 is an inhibitory T-cell surface receptor that promotes self-tolerance by suppressing T-cell activation. PD-L1, as the PD-1 ligand, is often overexpressed in tumor cells (7). Interaction between PD-1 and andPD-L1 is known to significantly inhibit antitumor immunity in T-cells, leading to immune evasion and resistance (8, 9). Immune checkpoint inhibitors targeting the PD-1/PD-L1 axis block the negative regulatory pathway, reactivating T cells to exert potent immune responses (10).

In clinical practice, a proportion of patients receiving anti-PD-1/PD-L1 inhibitors did not experience survival benefits. Approved biomarkers, including PD-L1 expression level, tumor mutational burden (TMB), and mismatch repair (MMR)/microsatellite instability (MSI), all have their own limitations (11). It is critical to explore non-invasive, cost-effective and easily accessible biomarkers for anti-PD-1/PD-L1 treatment. The peripheral hematological parameters of inflammation have been reported as prognostic biomarkers in patients with stage IV NSCLC and those receiving immunotherapy (12–14). Higher neutrophil-to-lymphocyte ratio (NLR) was associated with poor prognosis in advanced NSCLC patients receiving anti-PD-1 inhibitors (12), while higher NLR, higher platelet-to-lymphocyte ratio (PLR), and lower lymphocyte-to-monocyte ratio (LMR) at baseline were associated with poorer OS (13). Derived neutrophil-to-lymphocyte ratio (dNLR) was associated with lack of response to nivolumab (14). Nonetheless, few studies have investigated peripheral cell counts, including absolute neutrophil count (ANC), absolute lymphocyte count (ALC), absolute monocyte count (AMC), absolute eosinophil count (AEC), absolute platelet count (APC) and absolute leukocyte count (ALeC), as potential biomarkers for clinical outcome (15). In addition, although nivolumab and pembrolizumab were generally considered interchangeable (6, 16), they bind to different epitopes on the receptor and exhibit different affinities (6). The differences in outcome and associated biomarkers between these two agents remain unknown. In the current retrospective study, we aimed to investigate the clinical efficacies of pembrolizumab as first-line therapy as well as nivolumab monotherapy as subsequent-line treatment and to evaluate the correlations between hematologic parameters and clinical outcomes of different anti-PD-1 therapies in advanced NSCLC patients.

## Materials and methods

### Patients

In this study, 79 patients with advanced (stage IIIB to IV) NSCLC who were treated with pembrolizumab as first-line therapy (pembrolizumab group) and 82 patients who were treated with nivolumab monotherapy as subsequent-line therapy (nivolumab group) at the Fudan University Shanghai Cancer Center between January 2016 and March 2021 were included in the analysis. Peripheral hematologic parameters before the treatment were collected. Last follow-up was conducted in November 2021. The study was performed according to protocols approved by the institutional review board of the Fudan University Shanghai Cancer Center.

### Survival assessments

Progression-free survival (PFS) was measured from the time of treatment initiation to clinical or radiographic progression or death from any cause. Overall survival (OS) was measured from the time of treatment initiation to death from any cause. Patients without documented clinical or radiographic disease progression or who were still alive were censored on the date of the last follow-up.

### PD-L1 expression analysis

Tissue samples were fixed in 10% formaldehyde, embedded in paraffin, cut into 4 to 7 μm sections and attached to glass slides. Tissues were deparaffinized with xylene, hydrophilized and unmasked following routine immunohistochemical procedure. The commercial PD-L1 immunohistochemistry assay (clone 22C3; DAKO Autostainer Link48; RTU) was used to assess PD-L1 status in line with the manufacturer’s instructions. PD-L1 score was defined as tumor proportion score (TPS) with the following criteria: percentage of viable tumor cells showing partial or complete membrane PD-L1 staining at any intensity (only membranous staining).

### Statistical analysis

Patients’ clinical characteristics were compared using Fisher’s exact test for discrete variables and the Wilcoxon rank sum test for continuous variables. The optimal cutoff value for hematologic parameters was assessed using X-Tile (17) software. Kaplan-Meier analyses of PFS and OS were performed, with the log-rank test used to calculate the P value. The hazard ratio (HR) and 95% confidence interval (CI) were calculated with the Cox proportional hazard model. Parameters with P values less than 0.1 in the univariate analysis were selected for multivariate analysis. Clinical factors, including age (≥60 vs. < 60 years), brain metastatic (yes vs. no) or liver metastatic (yes vs. no) were used as covariates in multivariate Cox proportional hazard regression analysis. Receiver operating characteristic (ROC) curve was used to analyze the predictive role of factors for progression status. A two-sided P-value below 0.05 was considered significant. All statistical tests were conducted in R software (version 3.6.1).

## Results

### Patient characteristics

The baseline characteristics of all patients were summarized in **Table 1**. The median age at diagnosis was 63 years old (range: 27 to 78 years), 82.6% (133/161) of the patients were males, 97.5% (157/161) had ECOG PS score of 1, 75.1% (121/161) were stage IV, 85.7% (138/161) were no smoking, 50.9% (82/161) had one metastatic site, 75.9% (128/161) without radiation therapy and the common metastatic site at the pleura or bone occurred in 59.0% (95/161) patients. Of those with sufficient histologic or PD-L1 TPS information, 55.2% (84/152) were adenocarcinoma, 73.5% (50/68) were PD-L1 TPS < 1% (36.8%, 25/68) or > 50% (36.8%, 25/68). Except for ages, treatment regimen and line of treatment, all other characteristics at baseline were balanced in the pembrolizumab group and the nivolumab group. The median age at diagnosis was 64 years old (range: 32 to 78 years) in the pembrolizumab group and 61 years old (range: 27-77) in the nivolumab group. A higher percentage of patients in the pembrolizumab group were ≥60 years old than in the nivolumab group (73.4% *vs.* 57.3%, P=0.048). In the pembrolizumab group, all patients received first-line pembrolizumab treatment, with 81.01% received pembrolizumab combined with chemotherapy, 16.46% received pembrolizumab monotherapy and 2.5% received pembrolizumab combined with anti-vascular therapy. In the nivolumab group with subsequent-line treatment, 80.49% of the patients were treated with nivolumab monotherapy, 12.20% with nivolumab combined with ipilimumab, 6.1% with nivolumab combined with chemotherapy and 1.22% with nivolumab combined with anti-vascular therapy.

**Table 1:** Demographic and clinical characteristics of the study cohort.

	Pembrolizumab group (n = 79)	Nivolumab group (n = 82)	Total (n = 161)	P value
**Sex**				0.30
Male	68 (86.1%)	65 (79.3%)	133 (82.6%)	
Female	11 (13.9%)	17 (20.7%)	28 (17.4%)	
**Age**				0.046
≥60	58 (73.4%)	47 (57.3%)	105 (65.2%)	
<60	21 (26.6%)	35 (42.7%)	56 (34.8%)	
Median (range)	64 (32~78)	61 (27~77)	63(27-78)	
**Pathological subtype**				0.174
Adenocarcinoma	33 (41.8%)	51 (62.2%)	84 (52.2%)	
Squamous carcinoma	31 (39.2%)	29 (35.4%)	60 (37.3%)	
Others	7(8.9%)	1 (1.2%)	82 (50.9%)	
Unknown	8 (10.1%)	1 (1.2%)	9 (5.6%)	
**ECOG**				0.056
0	4 (5.1%)	0 (0.0%)	4 (2.5%)	
1	75 (94.9%)	82 (100%)	157 (97.5%)	
**Stage**				0.366
III	17 (21.5%)	23 (28.1%)	40 (24.8%)	
IV	62 (78.5%)	59 (72.0%)	121 (75.2%)	
**Smoking status**				0.178
Yes	8 (10.1%)	15 (18.3%)	23 (14.3%)	
No	71 (89.9%)	67 (81.7%)	138 (85.7%)	
**Metastatic site**				0.392
Bone	22 (27.9%)	25 (30.5%)	47 (29.2%)	
Liver	7 (8.9%)	9 (11.0%)	16 (9.9%)	
Brain	12 (15.2%)	14 (17.1%)	26 (16.2%)	
Adrenal glands	9 (11.4%)	3 (3.7%)	12 (7.5%)	
Pleura	27 (34.2%)	21 (25.6%)	48 (29.8%)	
**Number of metastasis sites**				0.7227
0	20 (25.3%)	23 (28.1%)	43 (26.7%)	
1	41 (51.9%)	41 (50.0%)	82 (50.9%)	
2	13 (16.5%)	16 (19.5%)	29 (18.0%)	
3	3 (3.8%)	2 (2.4%)	5 (3.1%)	
4	2 (2.5%)	0 (0.0%)	2 (1.2%)	
**PD-L1 status**				0.6996
<1%	14 (17.7%)	11 (13.4%)	25 (15.5%)	
1%~49%	10 (12.7%)	8 (9.8%)	18 (11.2%)	
>50%	17 (21.5%)	8 (9.8%)	25 (15.5%)	
Unknown	38 (48.1%)	55 (67.1%)	93 (57.8%)	
**Radiation therapy**				0.0514
Yes	11 (13.9%)	22 (26.8%)	33 (20.5%)	
No	68 (86.1%)	60 (73.2%)	128 (79.5%)	
**Treatment regimen**				< 0.001
Immune monotherapy	13 (16.46%)	66 (80.5%)	79 (49.1%)	
Immune and chemotherapy	64 (81.01%)	5 (6.1%)	69 (42.9%)	
Immune and anti-vascular	2 (2.53%)	1 (1.2%)	3 (1.9%)	
Immune and immune	0 (0.00%)	10 (12.2%)	10 (6.2%)	
**Treatment line**				< 0.001
1^st^	79 (100%)	0 (0.0%)	79 (49.1%)	
≥2^nd^	0 (0.00%)	82 (100.0%)	82 (50.9%)	

ECOG PS, Eastern Cooperative Oncology Group performance- status; PD-L1 TPS, programmed death-1 tumor proportion score.

### Clinical outcomes

**Figure 1:**
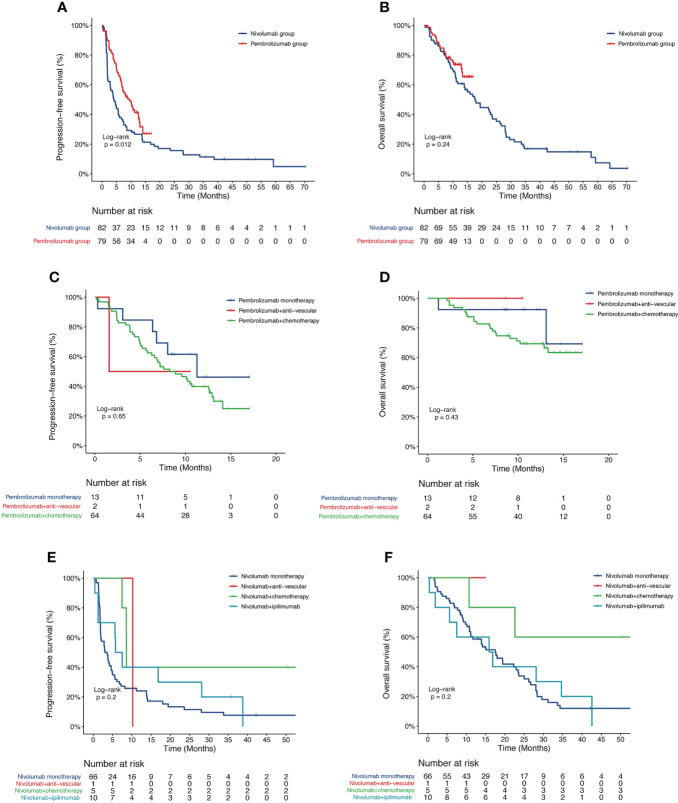
Kaplan-Meier curve of PFS and OS of 161 patients. **(A)** PFS and **(B)** OS of patients stratified with nivolumab and pembrolizumab; **(C)** PFS and **(D)** OS of patients stratified with pembrolizumab monotherapy, pembrolizumab plus anti-vascular therapy and pembrolizumab plus chemotherapy; **(E)** PFS and **(F)** OS of patients stratified with nivolumab monotherapy, nivolumab plus anti-vascular therapy, nivolumab plus chemotherapy and nivolumab plus ipilimumab therapy. PFS, progression-free survival; OS, overall survival.

**Figure 1** showed the PFS and OS of first-line pembrolizumab and subsequent-line nivolumab treatment. The mPFS was 9.6 months in the pembrolizumab group and 4.1 months in the nivolumab group (P=0.012). In pembrolizumab group, different treatment regimen showed no significant difference in PFS, with mPFS of 11.27 months, 8.27 months and 1.57 months in those with pembrolizumab monotherapy (13/79), pembrolizumab-chemotherapy (64/79) and pembrolizumab-anti-vascular therapy (2/79), respectively (P=0.65, **Figure 1C**. The mOS of three subgroups have not been reached (**Figure 1D**). Similarly, in the nivolumab group, no significant difference in PFS was found among patients with nivolumab monotherapy (66/82, mPFS=3.32 months), nivolumab combined with chemotherapy (5/82, mPFS=5 months), nivolumab combined with ipilimumab (10/82, mPFS=6.63 months) and nivolumab combined with anti-vascular (1/82, mPFS=10 months) subgroups (P=0.20, **Figure 1E**. The mOS were 17.5 months and 16.4 months in nivolumab monotherapy and nivolumab combined with ipilimumab subgroups, while the OS of the remaining two subgroups have not been reached (**Figure 1F**).

### Baseline hematologic parameters and outcome in the pembrolizumab group

**Figure 2:**
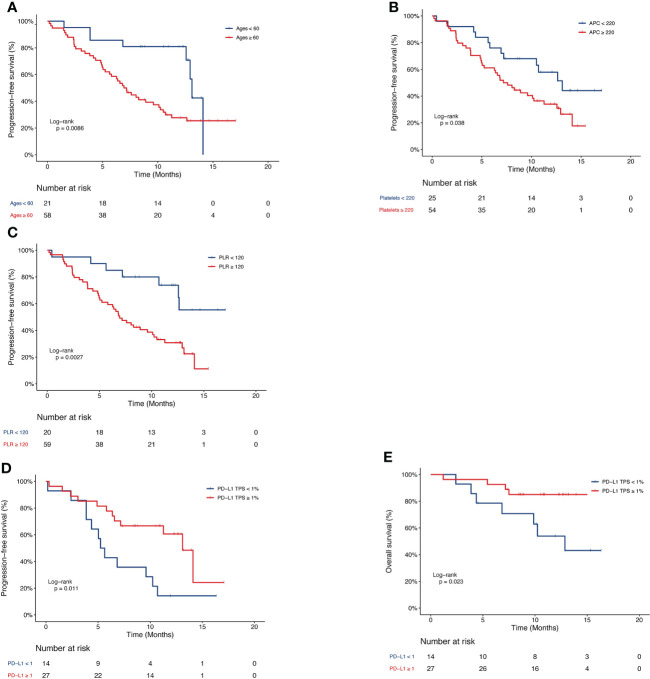
Kaplan-Meier curve of PFS or OS of patients in the pembrolizumab group. PFS of patients stratified by **(A)** age, **(B)** APC, **(C)** PLR and **(D)** PD-L1 TPS. **(E)** OS of patients stratified by PD-L1 TPS. PFS, progression-free survival; OS, overall survival; APC, absolute platelet count; PLR, platelet-to-lymphocyte ratio; PD-L1 TPS, programmed death-1 tumor proportion score.

X-tile software was used to determine the optimal cutoff value of hematologic parameters for survival analysis. The optimal cutoff value for parameters were 180 U/L of LDH, 10×10^9^/L of ALeC, 7×10^9^/L of ANC, 1.6×10^9^/L of ALC, 0.7×10^9^/L of AMC, 0.45×10^9^/L of AEC, 220×10^9^/L APC of, 4.5 of NLR, 2.8 of dNLR, 120 of PLR and 2.4 of LMR. Univariate and multivariate analyses were performed to explore the association between the survival outcomes and the hematologic parameters, as well as other relevant clinical characteristics. In univariate analysis, negative associations were found between PFS and baseline factors, including age, brain metastatic, ALeC, ANC, AMC, AEC, APC, LDH, NLR, dNLR and PLR, whereas PD-L1 expression, ALC and LMR exhibited positive associations with PFS (**Supplementary Table 1**). Other clinical characteristics, such as metastasis to different sites, including the bone, liver, adrenal glands and pleura, number of metastatic sites and radiation therapy, showed no association with PFS. Multivariate analysis confirmed that lower age, higher PD-L1 expression, lower APC and lower PLR were independent prognostic factors of longer PFS (HR = 7.32, P = 0.004; HR = 0.26, P = 0.007; HR = 5.25, P = 0.009; HR = 10.72, P = 0.012). KM Kaplan-Meier estimates of PFS probabilities according to ages, APC, PLR and PD-L1 TPS were shown in **Figure 2**. The median PFS were 13.1 months and 7.1 months for ages < 60 vs. ≥ 60 years, respectively (**Figure 2A**). The median PFS were 10.5 months and 5.2 months for APC < 220 vs. ≥ 220 ×10^9^/L, respectively (**Figure 2B**). The median PFS were 12.6 months and 5.2 months for PLR < 120 vs. ≥ 120, respectively (**Figure 2C**). The median PFS were 5.4 months and 13.1 months for PD-L1 TPS < 1% vs. ≥ 1%, respectively (**Figure 2D**). In both univariate (**Supplementary Table 1**) and multivariate (HR=0.16, P=0.013, **Table 2**) analyses, higher PD-L1 expression remained significantly associated with longer OS. The median OS were 12.9 months and not reached for PD-L1 TPS < 1% vs. ≥ 1%, respectively (**Figure 2E**). Correlation of OS with other factors, including LDH, ALeC, ANC, ALC, AMC, AEC, APC, NLR, dNLR, PLR and LMR was not observed in multivariate analysis (**Supplementary Table 1**).

**Table 2:** Estimates for hazard ratios for progression-free survival and overall survival in the pembrolizumab group.

Factors	PFS		OS	
	Multivariate analysis		Multivariate analysis	
	HR (95% CI)	P value	HR (95% CI)	P value
Age (≥60 vs <60)	7.32 (1.90~28.21)	0.004		
PD-L1 status (≥1% vs <1%)	0.26 (0.10~0.70)	0.007	0.16 (0.04~0.68)	0.013
APC (≥220 vs <220)	5.25 (1.51~18.25)	0.009		
PLR (≥120 vs <120)	10.72 (1.67~68.81)	0.012		

HR, hazard ratios; CI, confidence interval; PFS, progression-free survival; OS, overall survival; PD-L1 TPS, programmed death-1 tumor proportion score; APC, absolute platelet count; PLR, platelet-to-lymphocyte ratio.

### Baseline hematologic parameters and outcome in the nivolumab group

**Figure 3:**
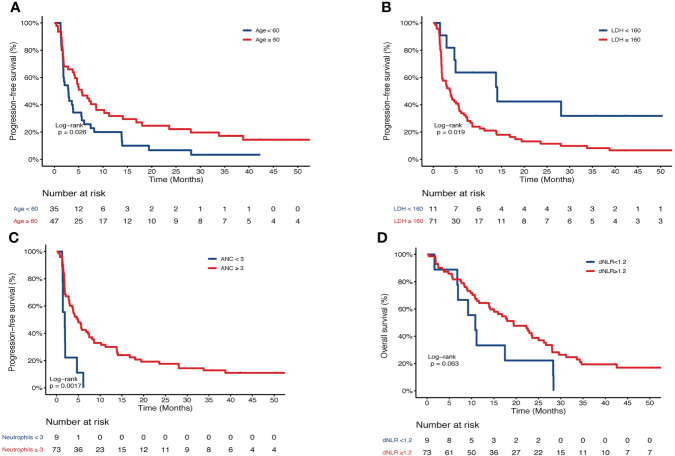
Kaplan-Meier curve of PFS or OS of patients in the nivolumab group. PFS of patients stratified by **(A)** age, **(B)** LDH, and **(C)** ANC. **(D)** OS of patients stratified by dNLR. PFS, progression-free survival; OS, overall survival; LDH, lactate dehydrogenase; ANC, absolute neutrophil count.

Similarly, the levels of LDH, ALeC, ANC, NLR, dNLR, PLR and LMR were dichotomized at 160 U/L, 11×10^9^/L, 3×10^9^/L, 1.5, 1.2, 160 and 1.6, respectively. Univariate and multivariate analyses were performed to investigate the survival outcomes in association with these clinical parameters. Based on univariate analysis, age and liver metastasis status were also included in the multivariate analysis. Other clinical factors, including smoking status, metastatic site, number of metastases and radiation therapy were not included in the multivariate analysis due to insignificant P values in the univariate analysis (**Supplementary Table 2**). Specifically, univariate analysis revealed significant associations between PFS and the factors such as age, liver metastasis, ALeC, LDH, ANC, NLR, dNLR and PLR (**Supplementary Table 2**). Further multivariate analyses confirmed that higher LDH (HR=2.88, P=0.013) was an independent indicator for poorer PFS. Age (HR=0.52, P=0.014) and higher ANC (HR=0.20, P=0.004) were independent prognostic factors for longer PFS (**Table 3**). Meanwhile, univariate analysis revealed significant association between OS and factors such as ALeC, PLR and LMR (HR = 0.16, P = 0.046; HR = 1.75, P = 0.029; HR = 0.35, P = 0.041, **Supplementary Table 2**). Multivariate analyses of OS suggested that high dNLR (HR=0.46, P=0.014) was an independent factor for longer OS (**Table 3**). KM estimates of PFS probabilities or OS probabilities according to ages, LDH, ANC and dNLR were showed in **Figure 3**. The median PFS were 2.9 months and 5.6 months for ages < 60 vs. ages ≥ 60 years, respectively (P =0.026, **Figure 3A**). The median PFS were 14.1 months and 3.8 months for LDH < 160 vs. ≥ 160 U/L, respectively (P =0.019, **Figure 3B**). The median PFS were 1.9 months and 4.9 months for ANC < 3 vs. ≥ 3 ×10^9^/L, respectively (P =0.017, **Figure 3C**). The median OS were 10.8 months and 19.4 months for dNLR < 1.2 vs. dNLR ≥ 1.2, respectively (P =0.063, **Figure 3D**).

**Table 3:** Estimates for hazard ratios for progression-free survival and overall survival in the nivolumab group.

Factors	PFS		OS	
	Multivariate analysis		Multivariate analysis	
	HR (95% CI)	P value	HR (95% CI)	P value
Age (≥60 vs <60)	0.52 (0.31~0.88)	0.014		
LDH (≥160 vs <160)	2.88 (1.25~6.63)	0.013		
ANC (≥3 vs <3)	0.20 (0.07~0.60)	0.004		
dNLR (≥1.2 vs <1.2)			0.46 (0.22~0.99)	0.049

HR, hazard ratios; CI, confidence interval; PFS, progression-free survival; OS, overall survival; LDH, lactate dehydrogenase; ANC, absolute neutrophil count; dNLR, derived neutrophil-to-lymphocyte ratio.

### The predictive ability of change in hematologic parameters for progressive disease

**Figure 4:**
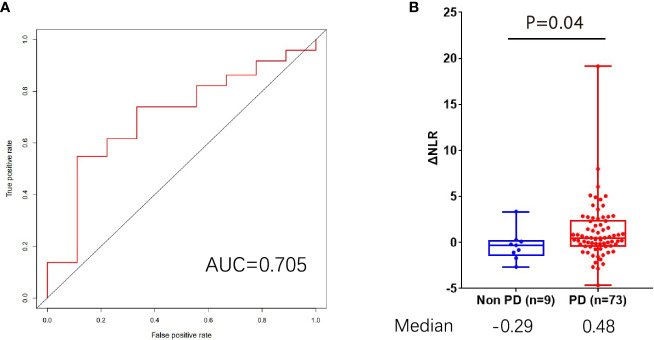
The predictive role of △NLR for progressive disease. **(A)** AUC analysis of the △NLR. **(B)** Distribution of △NLR value in PD and non-PD groups. PD, progressive disease; AUC, areas under the receiver operating characteristic curve; NLR, neutrophil-to-lymphocyte ratio.

Patients were further divided into **progressive disease (**PD) group and non-PD group by the time of last follow-up. The change (△ value) in the parameter levels was calculated and receiver operating characteristic (ROC) curve of △value was conducted. In nivolumab group, the areas under the ROC curve (AUC) of △NLR was 0.705 (**Figure 4A**), and the difference between PD and non-PD patients was significant (median: 0.48 vs. -0.29, P=0.04, Wilcoxon test, **Figure 4B**). The areas under the ROC curve of all other hematologic parameters were all under 0.7 in both groups.

## Discussion

Currently, PD-L1 expression level in the tumor tissue is the best-accepted biomarker for treatment efficacy of anti-PD-1 or PD-L1 inhibitors in patients with advanced NSCLC. However, identification of additional biomarkers is important because PD-L1 expression status was not available for a proportion of patients due to reasons such as the lack of tumor tissue, high risk of biopsy, relatively high detection costs and the limited predictive accuracy. The advantage of using peripheral blood over tumor tissue as a source of biomarker is obvious. In previous studies, baseline counts of peripheral blood cells such as ANC, ALC, AEC as well as hematologic parameters, including NLR, LMR, PLR and LDH, have been found to be associated with outcome in patients with NSCLC (12, 13, 15, 18, 19). There has been a limited number of previous studies that correlated blood biomarkers and prognosis, most without taking into consideration the difference in immunotherapy regimens and therapeutic lines. Thus, potential blood biomarkers for immune checkpoint inhibitors (ICIs) in lung cancer have remained unclear. In our study, we specifically analyzed 161 patients who received first-line pembrolizumab or subsequent-line nivolumab monotherapy to eliminate potential confounding factors.

KEYNOTE-021 has indicated first-line pembrolizumab plus pemetrexed-carboplatin significantly improves the clinical efficacy regardless of PD-L1 status in advanced non-squamous NSCLC (20). However, we found that PD-L1 TPS (≥1%) was an independent prognostic factor for PFS and OS in first-line pembrolizumab treatment. The prognostic value of PD-L1 TPS should be interpreted with caution here due to our small sample size. Our study also indicated the potential role of PD-L1 expression level, APC, PLR, LDH, ANC and dNLR for prognosis and the necessity of daily monitoring during treatment.

As the treatment line and regimen were significantly different between the pembrolizumab and nivolumab group (both P < 0.05), we analyzed the correlations between hematologic parameters and anti-PD-1 outcome in the two groups separately. Overall, the hematologic biomarkers identified in the pembrolizumab group were consistent with the results reported in previous studies, while those in the nivolumab group were different from previous studies (15). Neutrophil was important in tumorigenesis, metastasis and angiogenesis through recruitment into the tumor stroma (21, 22), and low ANC in baseline peripheral blood was reported to be associated with favorable prognosis for nivolumab monotherapy (15). By contrast, higher baseline neutrophils were beneficial for PFS in our multivariate analysis in the nivolumab subgroup, which may be due to the fact that our patients received nivolumab as a subsequent-line therapy. Consequently, potential drug interactions might have impaired cell-mediated immunity, and ultimately influencing the efficacy of anti-PD-1 treatment and inflammatory parameters (23–25). Although the prognostic role of NLR was not observed in our cohort, △NLR was a potential biomarker for PD/non-PD prediction, which was consistent with previous studies (12).

There were some limitations for the present study. This was a single center retrospective study and the OS data was immature in the pembrolizumab group. Although univariate and multivariate analyses between biomarkers and OS would require longer clinical follow-up, our results support the clinical relevance of hematologic parameters and provide reference for similar studies in the future. In addition, we recognize the limitation of not being able to analyze the correlation between treatment history and immunotherapy efficacy in the nivolumab group. Indeed, several studies have reported that steroids therapy (23), antibiotic use (24) and proton pump inhibitors (25) affect the number of immune cell and inflammation-related cells in blood. Given the retrospective nature of the present study, we were unable to obtain sufficient information on treatment of patients, and we would focus on this in future investigations. Finally, the methods for determination of the threshold of hematologic parameters include ROC curve analysis (12, 26), X-tile (18) and percentile (27). The optimal cutoff for hematologic parameters was not fully consistent among existing reports (28, 29). Notably, the threshold for NLR has been reported to be in the range of 3-5 (28), which was consistent with the NLR threshold used in this study. In addition, the cutoff for PLR has exhibited a wide range from 106 to 300 (29), and the cutoff in the present study was within this range. Although these studies, along with our research, have investigated the prognostic value of inflammatory parameters, the retrospective design is limited in providing reliable clinical evidence. Therefore, the cutoffs for the potential biomarkers screened in the present study need to be validated in future prospective studies of anti-PD-1/PD-L1 treatment.

Further large prospective studies are needed to verify the prognostic value of these markers. Nevertheless, several useful prognostic biomarkers in the peripheral blood for both the pembrolizumab and nivolumab subgroups were identified in this study. Our findings provide information for future similar studies and help guide clinical management for advanced NSCLC patients receiving anti-PD-1 inhibitor treatment.

## Data availability statement

The original contributions presented in the study are included in the article/**Supplementary Material**. Further inquiries can be directed to the corresponding author.

## Ethics statement

The studies involving human participants were reviewed and approved by Fudan University Shanghai Cancer Center. The patients/participants provided their written informed consent to participate in this study.

## Author contributions

Conception and design: XZ and JW; Provision of study material or patients: XZ, XW, HY, HW, SS, ZH, and JW; Collection and/or assembly of data: CL and JZ; Data analysis and interpretation: XZ, JZ, YS, and JW; Manuscript writing: CL, YS, and JW; Final approval of manuscript: XZ, XW, HY, HW, SS, ZH, CL, JZ, YS, and JW.

## Acknowledgments

We would like to thank all the patients and family members who gave their consent on presenting the data in this study, as well as the investigators and research staff involved.

## Conflict of interest

Authors CL, JZ, and YS were employed by Nanjing Geneseeq Technology Inc., China.

The remaining authors declare that the research was conducted in the absence of any commercial or financial relationships that could be construed as a potential conflict of interest.

## Publisher’s note

All claims expressed in this article are solely those of the authors and do not necessarily represent those of their affiliated organizations, or those of the publisher, the editors and the reviewers. Any product that may be evaluated in this article, or claim that may be made by its manufacturer, is not guaranteed or endorsed by the publisher.
