# Supplementary material for: Prognostic value of hematologic parameters in advanced non-small cell lung cancer patients receiving anti-PD-1 inhibitors
Source: Front Immunol. 2022 Oct 20;13:1003581. doi: 10.3389/fimmu.2022.1003581 (PMC9631308; doi:10.3389/fimmu.2022.1003581)
Supplement: Supplementary file 1 [file Table_1.docx]

| Factors | PFS | | OS | |
| --- | --- | --- | --- | --- |
|  | Univariate analysis | | Univariate analysis | |
|  | HR (95% CI) | P value | HR (95% CI) | P value |
| Age (≥60 vs <60) | 2.66(1.25~5.69) | 0.009 | 2.50(0.74~8.43) | 0.125 |
| Smoking (yes vs. no) | 1.22(0.52~2.90) | 0.641 | 1.50(0.44~5.08) | 0.512 |
| Histology (adenocarcinoma vs. squamous carcinoma) | 1.23(0.655~2.32) | 0.516 |  |  |
| Stage (IV vs. III) | 1.44(0.72~2.89) | 0.3 | 2.06(0.61~6.94) | 0.233 |
| Brain metastasis (yes vs. no) | 2.22(1.10~4.49) | 0.023 | 1.89(0.82~4.38) | 0.129 |
| Bone metastasis (yes vs. no) | 1.09(0.59~2.02) | 0.79 | 1.75(0.65~4.72) | 0.263 |
| Liver metastasis (yes vs. no) | 1.18(0.46~2.99) | 0.732 | 0.66(0.15~2.83) | 0.569 |
| Adrenal glands metastasis (yes vs. no) | 1.72(0.77~3.85) | 0.178 | 2.06(0.69~6.15) | 0.186 |
| Pleura metastasis (yes vs. no) | 0.83(0.45~1.52) | 0.543 | 1.53(0.67~3.49) | 0.309 |
| One metastatic sites (yes vs. no) | 1.70(0.87~3.33) | 0.116 | 2.67(0.79~9.00) | 0.099 |
| Two metastatic sites (yes vs. no) | 1.29(0.95~1.76) | 0.098 | 1.54(1.06~2.35) | 0.036 |
| Three metastatic sites (yes vs. no) | 1.12(0.79~1.57) | 0.527 | 1.02(0.63~1.66) | 0.943 |
| Radiation therapy (yes vs. no) | 0.77(0.33~1.82) | 0.554 | 0.87(0.26~2.93) | 0.82 |
| PD-L1 TPS (≥1% vs <1%) | 0.36(0.16~0.82) | 0.011 | 0.26(0.08~0.91) | 0.023 |
| LDH (≥180 vs <180) | 1.33(0.76~2.3) | 0.314 | 2.43(1.00~5.91) | 0.043 |
| ALeC (≥10 vs <10) | 2.57(1.36~4.85) | 0.003 | 3.62(1.56~8.38) | 0.001 |
| ANC (≥7 vs <7) | 2.85(1.57~5.18) | <0.001 | 3.26(1.43~7.46) | 0.003 |
| ALC (≥1.6 vs <1.6) | 0.53(0.30~0.95) | 0.029 | 0.49(0.21~1.15) | 0.092 |
| AMC (≥0.7 vs <0.7) | 1.95(1.12~3.41) | 0.017 | 2.55(1.12~5.83) | 0.022 |
| AEC (≥0.45 vs <0.45) | 3.27(1.36~7.86) | 0.005 | 5.85(2.13~16.01) | 0.0001 |
| APC (≥220 vs <220) | 1.97(1.03~3.79) | 0.038 | 2.53(0.86~7.47) | 0.082 |
| NLR (≥4.5 vs <4.5) | 3.84(2.12~6.94) | <0.001 | 5.80(2.49~13.53) | <0.001 |
| dNLR (≥2.8 vs <2.8) | 2.89(1.59~5.27) | <0.001 | 3.57(1.57~8.14) | 0.001 |
| PLR (≥120 vs <120) | 3.23(1.44~7.24) | 0.003 | 4.22(0.99~18.06) | 0.035 |
| LMR (≥2.4 vs <2.4) | 0.28(0.16~0.50) | <0.001 | 0.20(0.08~0.50) | 0.0001 |

Supplementary Table 1. Univariate analyses of progression-free survival and overall survival in pembrolizumab group

HR, hazard ratios; CI, confidence interval; PFS, progression-free survival; OS, overall survival; PD-L1 TPS, programmed death-1 tumor proportion score; LDH, lactate dehydrogenase; NLR, neutrophil-to-lymphocyte ratio; dNLR, derived neutrophil-to-lymphocyte ratio; PLR, platelet-to-lymphocyte ratio; LMR, lymphocyte-to-monocyte ratio; ALeC, absolute leukocyte count; ANC, absolute neutrophil count; ALC, absolute lymphocyte count; AMC, absolute monocyte count; AEC, absolute eosinophil count; APC, absolute platelet count.
