# Supplementary material for: Prognostic value of hematologic parameters in advanced non-small cell lung cancer patients receiving anti-PD-1 inhibitors
Source: Front Immunol. 2022 Oct 20;13:1003581. doi: 10.3389/fimmu.2022.1003581 (PMC9631308; doi:10.3389/fimmu.2022.1003581)
Supplement: Supplementary file 2 [file Table_2.docx]

Supplementary Table 2. Univariate analysis of progression-free survival and overall survival in the nivolumab group

| Factors | PFS | | OS | |
| --- | --- | --- | --- | --- |
|  | Univariate analysis | | Univariate analysis | |
|  | HR (95% CI) | P value | HR (95% CI) | P value |
| Age (≥60 vs <60) | 0.59(0.37~0.94) | 0.026 | 0.79(0.48~1.29) | 0.338 |
| Smoking (yes vs. no) | 0.80(0.44~1.46) | 0.466 | 0.59(0.30~1.17) | 0.126 |
| Stage (IV vs. III) | 1.55(0.92~2.63) | 0.1 | 1.24(0.70~2.18) | 0.457 |
| Brain metastasis (yes vs. no) | 1.46(0.792~2.67) | 0.224 | 0.92(0.49~1.73) | 0.789 |
| Bone metastasis (yes vs. no) | 1.21(0.74~2.00) | 0.445 | 1.32(0.78~2.26) | 0.3 |
| Liver metastasis (yes vs no) | 2.37(1.16~4.83) | 0.014 | 1.74(0.79~3.84) | 0.163 |
| Adrenal glands metastasis (yes vs. no) | 1.45(0.46~4.65) | 0.525 | 0.85(0.27~2.73) | 0.785 |
| Pleura metastasis (yes vs. no) | 0.92(0.54~1.57) | 0.752 | 0.85(0.46~1.55) | 0.587 |
| One metastatic sites (yes vs. no) | 1.53(0.90~2.59) | 0.113 | 1.20(0.68~2.12) | 0.537 |
| Two metastatic sites (yes vs. no) | 1.18(0.89~1.55) | 0.249 | 1.11(0.83~1.49) | 0.489 |
| Three metastatic sites (yes vs. no) | 1.05(0.66~1.69) | 0.827 | 0.98(0.61~1.57) | 0.936 |
| Radiation therapy (yes vs. no) | 1.26(0.76~2.1) | 0.366 | 0.96(0.55~1.67) | 0.88 |
| PD-L1 TPS (≥1% vs <1%) | 1.23(0.49~3.06) | 0.663 | 1.18(0.48~2.88) | 0.718 |
| LDH (≥160 vs <160) | 2.49(1.13~5.45) | 0.019 | 1.95(0.84~4.53) | 0.116 |
| ALeC (≥11 vs <11) | 0.16(0.02~1.21) | 0.045 | 0.16(0.02~1.22) | 0.046 |
| ANC (≥3 vs <3) | 0.33(0.16~0.68) | 0.002 | 0.69(0.34~1.41) | 0.309 |
| NLR (≥1.5 vs <1.5) | 0.37(0.14~0.922) | 0.026 | 1.05(0.42~2.62) | 0.924 |
| dNLR (≥1.2 vs <1.2) | 0.49(0.24~1.01) | 0.047 | 0.51(0.25~1.05) | 0.064 |
| PLR (≥160 vs <160) | 1.53(0.96~2.45) | 0.072 | 1.75(1.05~2.90) | 0.029 |
| LMR (≥1.6 vs <1.6) | 0.59(0.24~1.50) | 0.264 | 0.35(0.12~1.00) | 0.041 |

HR, hazard ratios; CI, confidence interval; PFS, progression-free survival; OS, overall survival; PD-L1 TPS, programmed death-1 tumor proportion score; LDH, lactate dehydrogenase; ALeC, absolute leukocyte count; ANC, absolute neutrophil count; NLR, neutrophil-to-lymphocyte ratio; dNLR, derived neutrophil-to-lymphocyte ratio; PLR, platelet-to-lymphocyte ratio; LMR, lymphocyte-to-monocyte ratio.
